# GLP-1 and GIP class drugs have neuroprotective properties in Alzheimer’s and Parkinson’s disease

**DOI:** 10.3389/fnins.2026.1906426

**Published:** 2026-08-14

**Authors:** Christian Hölscher

**Affiliations:** 1 Department of Neurology, Second Associated Hospital, Shanxi Medical University, Taiyuan, Shanxi, China; 2 Kariya Pharmaceuticals Ltd., Copenhagen, Denmark

**Keywords:** Alzheimer’s, BBB, CNS, GIP, GLP-1, incretins, Parkinson’s

## Abstract

Alzheimer’s and Parkinson’s disease are two CNS diseases with a large unmet need for disease-modifying therapies. Recently, two phase 3 clinical trials testing the GLP-1 analogue Semaglutide (Wegovy, Ozempic) in patients with Alzheimer’s disease did not show improvements. This failure should not have been a surprise, since it has been known for many years that Semaglutide does not cross the blood-brain barrier (BBB) and enters the brain only in very small amounts. It is designed to stay in the blood stream and has a very long half-life in the blood (168 h). The GLP-1 analogue Liraglutide (Victoza), in contrast, has a much shorter half-life (13 h), and a phase 2 clinical trial showed improvements in cognitive tests after 1 year of treatment. GLP-1 receptor agonists have been tested in clinical trials in Parkinson’s patients, and a similar picture emerged. Drugs that can cross the BBB well (exenatide, lixisenatide) show good protection, while a drug that cannot cross the BBB showed no effects (NLY01). Clearly, when treating CNS diseases, it is of importance to get the drug into the brain to ensure target engagement. This review will look at the clinical trials that have been conducted in more detail, describe the mode of action as derived from preclinical trials, and discuss novel strategies for getting GLP-1 receptor agonists into the brain to successfully treat CNS diseases.

## Introduction

Glucagon-like peptide 1 (GLP-1) and glucose-dependent insulinotropic polypeptide (GIP) are peptide hormones that play several roles in the body. After a meal, they are released in the intestine to signal availability of glucose and lipids (Baggio and Drucker, 2007; Müller et al., 2019; Müller et al., 2025). In addition, GLP-1 and GIP are expressed by neurons in the CNS and act as a neurotransmitter (Cork et al., 2015; Larsen and Holst, 2005; Müller et al., 2025; Nyberg et al., 2005; Nyberg et al., 2007; Vrang and Larsen, 2010). Furthermore, GLP-1 is expressed in microglia and astroglia and plays a role in regulating the chronic inflammation response. It acts as an anti-inflammatory cytokine in this context (Iwai et al., 2006; Kappe et al., 2012; Kopp et al., 2022; Ohshima, 2015; Vrang and Larsen, 2010).

In addition, GLP-1 and GIP act as growth factors. Their G-protein coupled receptors activate the classic growth factor second-messenger cascade of cAMP/PKA/CREB signaling to enhance gene expression that is related to cell metabolism, cell growth and repair. In addition, other signaling pathways such as PI3k/Akt/mTor/FOXO1/3a can be activated under specific conditions (Hölscher, 2014; Müller et al., 2019; Perry and Greig, 2003; Reich and Hölscher, 2022). They reverse the loss of growth-factor signaling in the brains of Alzheimer’s disease (AD) and Parkinson’s disease (PD) patients and normalize glucose utilization in the brain (Athauda and Foltynie, 2016; Athauda et al., 2019; Hölscher, 2025). In addition, GLP-1 and GIP reduces the chronic inflammation response in the brain, a key driver of AD and PD (Hölscher, 2025; Kopp et al., 2022). Importantly, incretins protect synaptic transmission that is impaired in AD and PD and prevent the elimination of synapses as seen in AD and PD (Hölscher, 2022).

## GLP-1 and GIP can cross the BBB

Glucagon-like peptide 1 (GLP-1) and GIP receptors are expressed throughout the CNS, but interestingly enough, there is a mismatch between GLP-1 release by neurons and the expression of the GLP-1 receptor. Neurons that are located in the lower brain stem project to only limited brain areas in the basal brain, but the receptor is expressed throughout the CNS, including the neocortex and the hippocampus (Cork et al., 2015; Trapp and Cork, 2015). This confused researchers, who wondered where the GLP-1 would come from that activates these receptors in the brain. The solution of this riddle is that GLP-1 and GIP can cross the BBB and activate receptors in all areas. It has been known for a long time that peptide hormones that indicate metabolic status can cross the BBB in order to signal the availability of energy to neurons (Kastin and Akerstrom, 2003; Kastin et al., 2002). Importantly, GLP-1 receptor agonists that are on the market to treat diabetes can enter the brain at various levels, ranging from good penetration to very little, depending on the design of the molecule (Hunter and Hölscher, 2012; Rhea et al., 2024; Salameh et al., 2020).

Recently, the interest in areas of the brain that express GLP-1 receptors and that are behind the BBB has increased. For example, the reward center in the ventral striatum contains GLP-1 receptors, and activating it reduces appetite (van Bloemendaal et al., 2015). It furthermore reduces the reward experienced from smoking, drinking alcohol or taking other drugs (Jerlhag, 2018; Marquez-Meneses et al., 2025). In addition, there are areas in the limbic system that express the GLP-1 receptor and GLP-1 receptor agonists can improve mood disorders (Mansur et al., 2017a; Russo and Nestler, 2013). An analysis of a patient cohort data base of people who took GLP-1 receptor agonists to treat diabetes even showed an improvement in schizophrenia (Xie et al., 2025); and clinical trials in patients with mood disorders showed some improvements (Mansur et al., 2017a,b). Furthermore, GLP-1 and GIP receptor agonists have neuroprotective effects in the brains of animal models of AD and PD if they are able to cross the BBB (Perry and Greig, 2003; Reich and Hölscher, 2022; Zhang and Hölscher, 2020).

## Which GLP-1 receptor agonist can cross the BBB?

**FIGURE 1:**
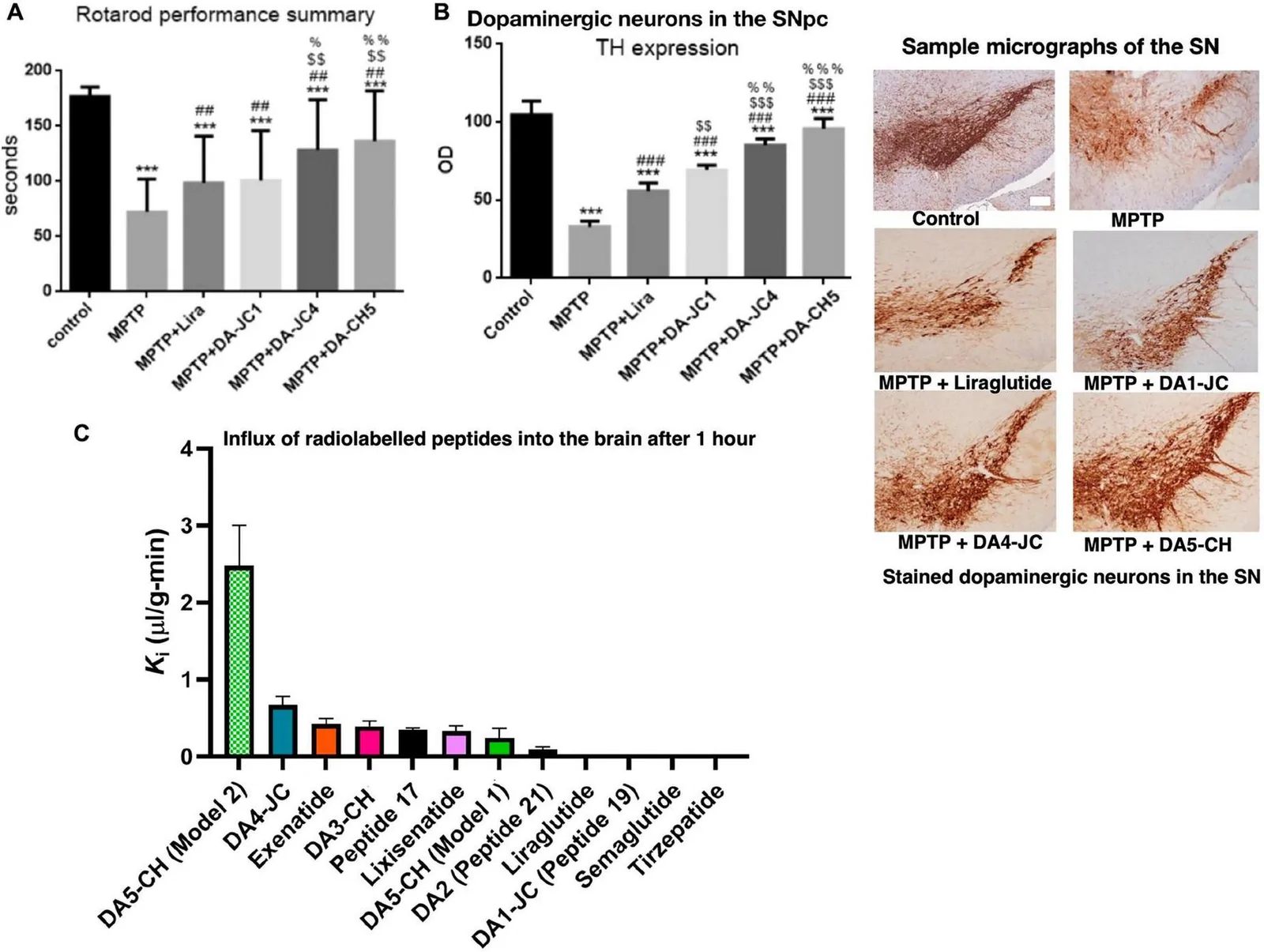
GLP-1 type peptide drugs show neuroprotection that correlates with their ability to cross the BBB. **(A)** Rotarod test of motor activity. DA4-JC and DA5-CH were most protective. ***p < 0.001 compared to controls; ##*p* < 0.01 compared to the MPTP group; $$*p* < 0.01 compared to the liraglutide + MPTP group. %*p* < 0.05 compared to the DA-JC1 + MPTP group, %%*p* < 0.01 compared to the DA1-JC + MPTP group. *N* = 10 per group. **(B)** Histology of dopaminergic neurons in the SN. Quantification of TH expression in neurons in the substantia nigra (SN). ***p < 0.001 compared to control group; ###*p* < 0.001 compared to MPTP group; $$*p* < 0.01 compared to the Liraglutide group; $$$*p* < 0.001 compared to the Liraglutide group; %%*p* < 0.01 compared to the DA-JC1 group; %%%*p* < 0.001 compared to the DA-JC1 group. Sample images of stained dopaminergic neurons in the SN. Scale bar = 200 Mm. Modified from Feng et al. (2018). **(C)** Rate constants (*K*i) of ^125^I/^14^C-radiolabeled peptide transport into whole brain tissue within 1 h after peripheral injection. Modified from Rhea et al. (2024). DA1-JC, a dual GLP-1/GIP agonist with a C16 fatty acid; DA2, a dual GLP-1/GIP agonist with a 40kDa pegylation; DA3-CH, a dual GLP-1/GIP agonist without modifications; peptide 17, a dual GLP-1/GIP agonist without modifications; DA4-JC, a dual GLP-1/GIP agonist with a poly-lys modification; DA5-CH, a dual GLP-1/GIP agonist with a Tat sequence modification.

Studies using radiolabelled GLP-1 receptor agonists showed that there are large differences between drugs in their ability to cross the BBB. In general, peptides that have not been modified to remain longer in the blood and that have a short half-life can cross the BBB best. Exenatide (Bydureon) and lixisenatide (Adlyxin) are such peptides that can cross the BBB relatively well (Salameh et al., 2020). Their half-lives in the blood are ca. 2.4 h for exenatide (Henry et al., 2014), and 3 h for lixisenatide (Barnett, 2011). Peptides that have been modified to stay longer in the blood to treat type 2 diabetes better are less able to cross the BBB. An example is liraglutide (Victoza), which has been modified with a C16 fatty acid to increase survival time in the blood to ca. 13 h. Its ability to cross the BBB is much reduced by this (Christensen et al., 2015; Knudsen and Lau, 2019; Salameh et al., 2020). Semaglutide (Ozempic), a modified GLP-1 peptide that has a C18 attached to it and an extended half-life in the blood of around 7 days. It is not able to cross the BBB in meaningful quantities, less than 0.1% reached the CSF in patients that took the drug (Johannsen et al., 2025; Knudsen and Lau, 2019; Rhea et al., 2024). The dual GLP-1/GIP receptor agonist tirzepatide has been modified by adding a C20 fatty acid to keep it in the blood for much longer. Its half-life in the blood is about 5 days (Chavda et al., 2022). Peptides that have been pegylated to enhance their survival time in the blood cannot cross the BBB very well either. A dual GLP-1/GIP receptor agonist that had been pegylated did not cross the BBB very well (Salameh et al., 2020; see Figure 1C). In conclusion, drugs that are designed to stay in the blood for a long time do not cross the BBB readily and are not ideal for treating CNS diseases. To enhance BBB penetration, we designed dual GLP-1/GIP receptor agonists that have a modification which enhances BBB penetration. For example, the dual agonist DA5-CH has a Tat sequence added (Feng et al., 2018; Hölscher, 2018). Tat sequences are positively charged amino acid sequences that are recognized by receptors that can take up the peptide and transport it across the BBB. Mainly, negatively charged proteoglycans and glycosaminoglycans that are expressed on the luminal membrane of endothelial cells attract positively charged peptides and import these peptides via adsorptive transcytosis (Hervé et al., 2008; Liu et al., 2008).

## Comparing different GLP-1 receptor agonists in animal models of AD or PD

Glucagon-like peptide 1 (GLP-1) receptor agonists have been tested in different animal models of CNS disease and have shown neuroprotective effects. Exendin-4 (exenatide) can enter the brain and protect neurons in animal models of AD (Isacson et al., 2011; Li et al., 2010), PD (Kim et al., 2009; Li et al., 2009). Lixisenatide can cross the BBB and shows neuroprotective effects in AD (Cai et al., 2014; Cai et al., 2017; Hunter and Hölscher, 2012; McClean and Hölscher, 2014b) or PD (Liu et al., 2015) animal models, too. In contrast, drugs that do not cross the BBB such as tirzepatide shows only limited effects in protecting the brain (Lingyu et al., 2026) or no effects at all (Forny Germano et al., 2024). A pegylated version of exendin-4 called NLY01 which does not cross the BBB readily, showed some limited effects in an animal model of AD (Park et al., 2021), or PD, where the drug did show good protective effects in a transgenic mouse model that has a leaky BBB, but limited effects in the MPTP mouse model of PD (Lv et al., 2021; Yun et al., 2018), and showed no effects in a clinical trial in PD patients (McGarry et al., 2024). The authors claim that NLY01 can cross the BBB and even claim that the modification of exendin-4 enhanced the uptake into the brain (Roy et al., 2025), but the data that they show refute this claim and show that the drug does not enter the brain in meaningful quantities in wild-type mice (Yun et al., 2018; see Supplementary Figure 2 of that paper). We tested the BBB penetration of NLY01 and could confirm that it does not enter the brain very well and showed only limited effects in the MPTP mouse model of PD (Lv et al., 2021). Pegylation of peptides has been developed as a technique to keep them suspended in the blood in order to treat type 2 diabetes better, not to cross the BBB better (Finan et al., 2015; Gault et al., 2008).

When comparing different GLP-1 receptor agonists head-to-head in animal models of disease, there is a clear trend of drugs that can cross the BBB easily being more effective in protecting the brain compared to drugs that do not. When comparing different dual GLP-1/GIP receptor agonists with liraglutide in a mouse model of PD, the dual agonists that can cross the BBB best were most effective, while a dual agonist that does not have a modification to enhance BBB crossing was not as effective. Liraglutide was the least effective drug in protecting dopaminergic neurons in the CNS (Feng et al., 2018; Yuan et al., 2017; see Figures 1A,B). When compared to semaglutide, a dual GLP-1/GIP agonist that can enter the brain better was more effective in protecting the brain in a PD animal model (Zhang et al., 2022). A different dual agonist that can enter the brain better was superior to liraglutide in a mouse model of AD (Maskery et al., 2020). When comparing the dual agonist DA5-CH with exendin-4 and tirzepatide, the dual agonist that can enter the brain was most effective, followed by exendin-4, and tirzepatide was least effective in protecting the brain in the 6-OHDA rat model of PD (Lingyu et al., 2026).

In conclusion, target engagement is key for GLP-1 receptor agonists to protect neurons. It is not sufficient to have the drug in the blood stream.

## Clinical trials testing GLP-1 receptor agonists in patients with AD or PD

As there are several GLP-1 receptor agonists on the market to treat diabetes, it was possible to repurpose these drugs and test them in patients with AD or PD. I will not discuss pilot studies that were underpowered and therefore could not show any statistically significant drug effects due to the fact that not enough patients were recruited. The results from such trials cannot be interpreted (Dei Cas et al., 2024; Gejl et al., 2016; Mullins et al., 2019).

## Clinical trials in AD patients

### Liraglutide reduced cognitive decline in AD patients

**FIGURE 2:**
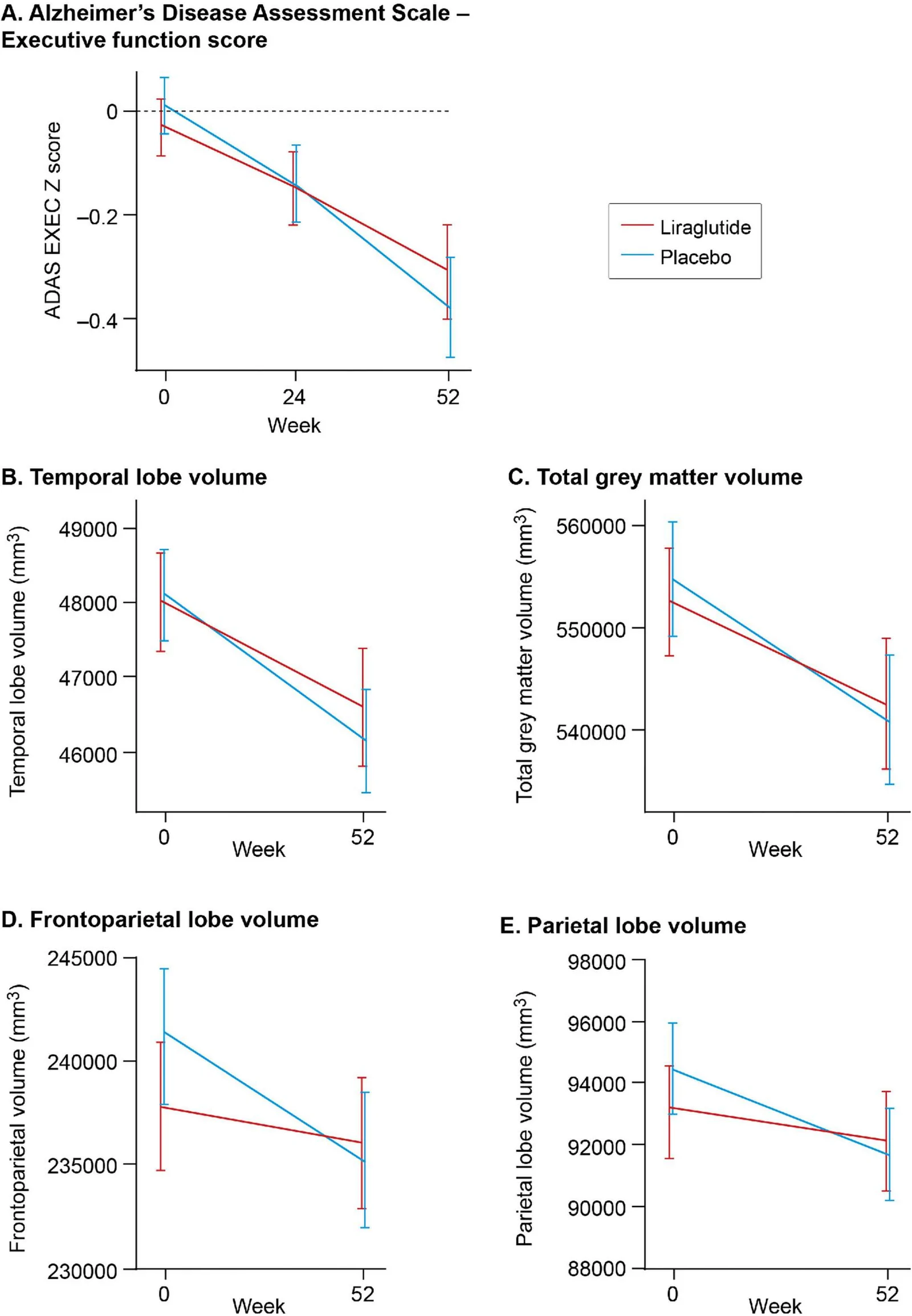
Treatment with liraglutide reduced disease progression in AD. **(A)** In the ADASexec cognitive test battery, drug-treated patients deteriorated more slowly than placebo-treated patients. The drug group started off with worse scores than the placebo group. After 6 months, both groups scored similar values, and after 12 months, the drug group scored better than the placebo group (*p* < 0.01). **(B–E)** MRI brain scans revealed that shrinking of key brain areas was significantly reduced by the drug. Modified from Edison et al. (2026).

As we were able to show good neuroprotective effects of liraglutide in animal models of AD such as improvement in cognition, reduction of the chronic inflammation response, reduced loss of synapses, improved synaptic transmission, and reduced levels of beta-amyloid (Batista et al., 2018; Han et al., 2013; Long-Smith et al., 2013; McClean and Hölscher, 2014a; McClean et al., 2011; Parthsarathy and Hölscher, 2013), we were able to secure funding to test liraglutide in a phase 2 trial in AD patients. In this study, 204 patients were randomly assigned to a drug or placebo group. Treatment was for 12 months (NCT01843075). In the ADASexec test battery, the liraglutide group deteriorated slower than the placebo group (*p* < 0.01). Other cognitive tests did not show a significant difference. In addition, brain shrinkage that is part of this disease was significantly reduced in key brain areas such as the prefrontal lobe and the parietal lobe that are linked to AD progression (see Figure 2). The uptake of glucose in the brain as measured by FDG-PET did not change. This biomarker was chosen as the primary endpoint (Edison et al., 2026).

This very first phase 2 trial testing a GLP-1 receptor agonist in AD patients already showed some positive outcomes and demonstrates that this approach is viable. Liragutide shows only limited BBB penetration, so other drugs that show better uptake into the brain may have superior protective effects.

### Two phase 3 clinical trials testing semaglutide in AD patients

Based on the success of the liraglutide study, two large studies testing semaglutide in AD patients were started (NCT04777396 and NCT04777409). Unfortunately, semaglutide has a very long half-life in the blood and does not cross the BBB in meaningful quantities. Measuring the amount of semaglutide in the CSF of patients after 12 weeks showed that less than 0.1% of the drug crossed the BBB as expressed as semaglutide concentration in blood plasma vs. CSF (see conference abstract (Johannsen et al., 2025)). The Evoke trial had 1855 patients and Evoke+ had 1953 patients at the starting point. The duration was 2 years with a possible extension of 1 year. Unsurprisingly, the trials did not show an improvement in a series of cognitive tasks after 2 years of treatment (Cummings et al., 2026). There were trends of improvements in several cognitive tests at week 130 and 156, but the overall effect was too little too late (Hölscher, 2026). An analysis of key biomarkers in the CSF after 78 weeks of treatment showed a reduction in some of the AD biomarkers: p-tau^181^; np-tau^205^; p-tau^217^; np-tau^181^; total tau levels; YKL-40, a marker of neuroinflammation. In addition, the biomarker neurogranin, which is associated with neurodegeneration, was reduced by drug treatment, also. Other biomarkers did not significantly change (Cummings et al., 2026). The fact that there was a shift in key AD biomarkers suggests that there was a limited drug effect that showed improvements in the pathology, but the shift was not big enough to translate in an improvement in the cognitive tests.

The limited improvements observed (even though semaglutide does not readily cross the BBB) support the notion that GLP-1 receptor agonists do have protective effects in the brain, but sufficient amounts of the drug need to get into the brain to obtain the full effect. To add to the story of semaglutide’s effect on AD, the analysis of large patient cohort data sets of over one million people who took semaglutide to treat diabetes showed a clear reduction in the number of patients who developed AD over the course of 3 years (Wang et al., 2024; Wang et al., 2025). This demonstrates that the drug does have some protective effects, but it is not enough to show a meaningful difference after 2 years in a clinical trial.

## Clinical trials in PD patients

### Exendin-4 (exenatide, byetta) showed good effects in a pilot study in PD patients

Exenatide was the first GLP-1 receptor agonist that had been licensed to treat diabetes (González et al., 2006). This made it possible to test exenatide in PD patients as the drug was already on the market to treat patients. A pilot study had been conducted to test exenatide (NCT01174810). This study did not have a placebo control as the organizers could not source it. As a first test, 44 patients were recruited, with 20 receiving the drug. Standard medication such as L-Dopa was taken by all patients as required. Type 2 diabetes was an exclusion factor. The average time from diagnosis with PD was 10 years, indicating that the patients were already quite advanced. The trial lasted for 12 months. At months 1, 6 and 12, the patients were assessed by the standard MDS-UPDRS part 3 (Movement Disorder Society-Unified Parkinson’s disease rating scale) tests. After the trial ended, patients were retested 3 months and 12 months later to evaluate if any improvements were still visible. The motor assessments were conducted by Neurologists who were blinded to treatment. Interestingly, patients were furthermore tested using the Mattis Dementia Rating Scale 2, a test battery to assess cognitive impairments. PD patients can develop cognitive impairments in the later stage of the disease (Matteau et al., 2011).

The drug treatment stopped disease progression, and patients scored similar values at months 1, 6, 12, 15 and 24. This is most remarkable as the drug was only administered in the first 12 months. The control group deteriorated significantly over the course of 2 years, as was expected for this disease. Interestingly enough, the patients that took the drug scored higher in the Mattis cognitive assessments, even after 24 months. In contrast, the control group got worse to a point where the Mattis dementia score 2 cannot measure any further deterioration (Llebaria et al., 2008). The difference could not be any clearer (Athauda and Foltynie, 2018; Aviles-Olmos et al., 2013, 2014).

The major drawback of this pilot study was that it did not have a placebo control. The MDS-UPDRS part 3 motor test battery requires that patients come to the hospital for an assessment by a Neurologist, which can illicit a placebo effect (de la Fuente-Fernández and Stoessl, 2002). The results did not convince critics, even though the protective drug effect remained stable for 12 months after the trial had stopped. A phase 2 trial with a placebo control was needed.

### A phase II study of exendin-4 (Exenatide, bydureon) in PD patients

Fortunately, the same group that ran the pilot study obtained funding to run a placebo-controlled, double blind phase II study (NCT01971242). In this study, 62 patients were tested and randomly assigned to the drug or placebo group. Exenatide was administered using the once-weekly formulation of exendin-4 (Bydureon). The average time period after PD diagnosis was around 6 years, shorter than in the pilot study. Patients took the standard medication for PD as required, and type 2 diabetes was an exclusion factor. When measuring peripheral insulin desensitization, 32% of patients were found to be partly desensitized as measured in HbA1c values in the blood of > 39. The trial lasted for 48 weeks, and patients were retested 12 weeks after the trial had stopped. In total, 62 patients were enrolled and randomly assigned, 32 to exenatide and 30 to placebo. After 60 weeks, off-medication scores on part 3 of the MDS-UPDRS had improved by 1⋅0 points (95% CI–2⋅6 to 0⋅7) in the exenatide group and worsened by 2⋅1 points (–0⋅6 to 4⋅8) in the placebo group, an adjusted mean difference of –3⋅5 points (–6⋅7 to –0⋅3; *p* = 0⋅0318) (Athauda et al., 2017). The drug group did not deteriorate over the course of the trial in motor tests, and even 3 months after the trial had stopped, the difference between groups was still significant. The placebo group deteriorated over time, as expected for this disease. The fact that the improvement in the drug group was still visible 12 weeks after the drug had been given last demonstrates that the drug is disease-modifying. Interestingly, cognitive performance also improved after drug treatment, mirroring the results of the pilot study (Athauda et al., 2018). In addition, when analysing biomarkers from exosomes that originate in the brain and can be found in the blood, insulin desensitization had been reduced by the drug (Athauda et al., 2019). This demonstrates that the drug is active in the brain and can reverse the disease-related insulin desensitization. The positive result is a proof of concept, demonstrating that GLP-1 receptor agonists have the potential to treat PD.

### A follow-up study, testing exendin-4 (exenatide, bydureon) in a larger patient group

Continuing the investigation of exenatide in PD patients, a larger study had been conducted by the same group. In this trial, 194 patients were randomly allocated to the drug or placebo group. The average time from diagnosis was 4.3 years, shorter than in previous studies, suggesting that disease progression may not be as advanced as in the earlier trials. Again, patients took the standard medication for treating PD as prescribed, and type 2 diabetes was an exclusion factor. In this cohort, the peripheral insulin desensitization in 17% of the patient cohort was found to be partly increased as measured in HbA1c values in the blood. These patients had not reached the threshold for type 2 diabetes, but were above the physiological baseline for HbA1c levels. The trial lasted for 96 weeks. There was no difference in the MDS-UPDRS part 3 motor test battery. Secondary outcomes were not significant either (NCT04232969) (Vijiaratnam et al., 2025).

Obviously, this result came as a bit of a shock. Further analyses showed that the responders to drug treatment were in the group that showed some insulin desensitization in the periphery. In the phase II study, 32% of patients had increased values of HbA1C levels > 39 (but were not diabetic). In this trial, however, only 17% of patients were in this category, see conference abstract (Foltynie, 2025). This suggests that GLP-1 receptor agonists do not work in all PD patients. It may be that there are separate subtypes of PD, some that develop peripheral insulin desensitization, and some that are not. Studies have shown that PD is not a homogenous disease, but that several subgroups exist that show large differences in brain pathology, disease progression, and insulin desensitization (Chahine et al., 2025). Another possibility is that the PD patients had not progressed in the disease far enough to develop peripheral insulin desensitization.

The lesson to be learned here is to stream PD patients for peripheral insulin desensitization to ensure a successful outcome. Future trials will have to incorporate this into their patient recruitment protocols.

### A phase II trial testing liraglutide (Victoza, saxenda) in PD patients

Liraglutide has been tested in a phase 2, double-blind, placebo-controlled clinical trial (NCT02953665). Patients were treated once-daily for 54 weeks. They received standard clinical PD medication as prescribed. The trial included 37 patients in the drug group and 18 in the placebo group. There were some positive outcomes and the drug group showed an improvement in the MDS-UPDRS part 2 tests. These assessments score everyday activities such as walking, talking, eating, conducting hobbies, getting dressed, and other measures. The overall Global MDS-UPDRS assessment scores were improved, too. There were other improvements in cognitive and memory tests, and in the PDQ-39 (quality of life) scores. However, in the MDS-UPDRS part 3 motor assessment, there was an improvement from baseline in both groups. The improvements in the placebo group are most likely due to the placebo effect. As these assessments are conducted in a hospital by a Neurologist, it is sensitive to the placebo effect. Therefore, the results of this primary endpoint cannot be interpreted, unfortunately. The results are available as a preprint (Hogg et al., 2023).

### A phase II study of lixisenatide (Lyxumia, adlyxin) in PD patients

Lixisenatide is a GLP-1 receptor agonist that can cross the BBB well, similar to exenatide (Rhea et al., 2024). It was of interest to test if this drug can improve PD disease progression, too. In a randomized, placebo-controlled, double-blind test, 156 patients received either drug or placebo for 1 year (NCT03439943). Two months after the trial had finished, patients were tested again to assess if any drug effect wore off after discontinuing drug treatment. The difference between groups in the MDS-UPDRS part III assessment was highly significant. The drug group did not deteriorate over the course of a year, while the placebo group did. Two months after the trial finished, the difference was still significant, demonstrating a disease-modifying effect (Meissner et al., 2024). The outcome of this trial mirrors those of the phase 2 Bydureon trial, and demonstrates that GLP-1 receptor agonists have the potential to treat PD patients, at least those that show peripheral insulin desensitization.

### A phase 2 clinical trial, testing NLY01 in PD patients

As mentioned before, a company developed a pegylated version of exenatide as a potential treatment for PD. The peptide is modified with a 40kDa pegylation, making it a large molecule ten times the original size. The advantage of pegylation is that it keeps the peptide in the blood stream for much longer. The company reported that the half-life in the blood improved from around 2 h (exenatide) to 88 h (pegylated exenatide) in non-human primates after pegylation (Yun et al., 2018). This means that the drug cannot leave the blood rapidly and cross the BBB, as claimed by the authors. The data presented clearly showed that NLY01 did not cross the BBB in wild-type mice in meaningful quantities (Yun et al., 2018; Supplementary Figure 2). It is therefore not surprising that the drug showed no effect in a phase 2 placebo-controlled clinical trial in the primary and secondary MDS-UPDRS test endpoints (NCT04154072). Drug treatment was for 36 weeks, and there were three groups of 85 patients each that received either placebo, low dose NLY01, or high dose NLY01 (McGarry et al., 2024).

### Developing GLP-1 receptor agonists that can cross the BBB

The available clinical evidence demonstrates that the ability of such peptides to cross the BBB is a key element for success. We therefore developed two dual GLP-1/GIP receptor agonists that have amino acid sequences added in order to enhance the transport across the BBB easier (Abdulhameed et al., 2024; Rhea et al., 2024; Salameh et al., 2020). The dual agonist DA5-CH has a Tat sequence added. Such sequences are recognized by cell surface receptors and enhance the transport across the cell membrane and the BBB by binding to receptors that can take up the peptide and transport it across the BBB (Hervé et al., 2008; Liu et al., 2008).

In a range of animal models of AD or PD, we tested these dual agonists and showed that they were more effective than the standard GLP-1 receptor agonists exendin-4, liraglutide, semaglutide, and tirzepatide that had been developed to treat diabetes (Feng et al., 2018; Lv et al., 2021; Maskery et al., 2020; Yang et al., 2022; Zhang et al., 2020, 2022). In a direct comparison between semaglutide and DA5-CH in the 6-OHDA rat model of PD, DA5-CH was superior in improving motor control, protecting the dopaminergic neurons in the SN and in reducing the inflammation response (Zhang et al., 2022). In addition, in a direct comparison between the dual agonist tirzepatide and DA5-CH in the same animal model, DA5-CH was better in protecting the dopaminergic neurons in the SN and in reducing the inflammation response (Lingyu et al., 2026). DA5-CH (KP405) has finished phase I clinical trials. It was safe to take in healthy aged subjects and in patients with PD. A study of biomarkers found in exosomes that originate in the brain showed that drug treatment improved biomarkers of inflammation (CD9, CD63, CD81, GRIN2a, NFkB) and of insulin signaling (Akt, IRS-1, mTOR, MAP Kinase) in the brain. Levels of alpha-synuclein were reduced, too. These results show target engagement of the dual agonist that had been injected once-daily subcutaneously and already showed improvements of key biomarkers in the CNS after day 1 (Eitan et al., 2026). A phase 2 trial in PD patients is in planning.

## Conclusion

The available clinical evidence demonstrates that GLP-1 receptor agonists that can enter the brain in meaningful quantities have the potential to reduce disease progression in AD and PD and perhaps even in other neurodegenerative disorders of the brain. In PD patients, an important factor is peripheral insulin desensitization that correlates with drug response and showed the best improvements (see Table 1). Further research is required to identify which patient subgroups at which state of disease progression are most likely to benefit from such drugs. The future is bright for the development of novel treatments that make a difference to patients without serious side effects.

**TABLE 1:** Summary of all clinical trials discussed in this review and the main outcomes.

Trial	Patient numbers	Half-life in the blood	BBB penetration	Outcomes
Phase 2 testing liraglutide in AD patients (NCT01843075)	204	13 h	Limited	Improvement in cognition, reduction in brain shrinkage
Phase 3 testing semaglutide in AD patients (NCT04777396 and NCT04777409)	Evoke 1855 Evoke+ 1953 (at start)	168 h	Poor	Small improvements in biomarkers, no improvements after 2 years
Pilot study testing exenatide in PD patients (NCT01174810)	44	2.4 h	Good	Improvement in motor tasks and cognitive tests
Phase 2 study testing exenatide in PD patients (NCT01971242)	62	2 weeks (Bydureon slow-release formulation)	Good	Improvement in motor tasks
Phase 3 study testing exenatide in PD patients (NCT04232969)	194	2 weeks (Bydureon slow-release formulation)	Good	No improvements
Phase 2 study testing lixisenatide in PD patients (NCT03439943)	156	3 h	Good	Improvement in motor tasks
Phase 2 study testing liraglutide in PD patients (NCT02953665)	37 drug, 18 placebo	13 h	Limited	Limited improvements, placebo effect in primary endpoint
Phase 2 study testing NLY01 in PD patients (NCT04154072)	255, 3 groups: high dose/ low dose/ placebo	88 h (non-human primates)	Poor	No improvements

## References

[B1] Abdulhameed N. Babin A. Hansen K. Weaver R. Banks W. Talbot K. et al. (2024). Comparing regional brain uptake of incretin receptor agonists after intranasal delivery in CD-1 mice and the APP/PS1 mouse model of Alzheimer’s disease. *Alzheimers Res. Ther.* 16:173. 10.1186/s13195-024-01537-1 39085976 PMC11293113

[B2] Athauda D. Foltynie T. (2016). Insulin resistance and Parkinson’s disease: A new target for disease modification? *Prog. Neurobiol.* 145-146 98–120. 10.1016/j.pneurobio.2016.10.001 27713036

[B3] Athauda D. Foltynie T. (2018). Protective effects of the GLP-1 mimetic exendin-4 in Parkinson’s disease. *Neuropharmacology* 136 260–270. 10.1016/j.neuropharm.2017.09.023 28927992

[B4] Athauda D. Gulyani S. Karnati H. Li Y. Tweedie D. Mustapic M. et al. (2019). Utility of neuronal-derived exosomes to examine molecular mechanisms that affect motor function in patients With Parkinson disease: A secondary analysis of the exenatide-PD trial. *JAMA Neurol.* 76 420–429. 10.1001/jamaneurol.2018.4304 30640362 PMC6459135

[B5] Athauda D. Maclagan K. Budnik N. Zampedri L. Hibbert S. Skene S. et al. (2018). What effects might exenatide have on non-motor symptoms in Parkinson’s disease: A post hoc analysis. *J. Parkinsons Dis.* 8 247–258. 10.3233/JPD-181329 29843254

[B6] Athauda D. Maclagan K. Skene S. Bajwa-Joseph M. Letchford D. Chowdhury K. et al. (2017). Exenatide once weekly versus placebo in Parkinson’s disease: A randomised, double-blind, placebo-controlled trial. *Lancet* 390 1664–1675. 10.1016/S0140-6736(17)31585-4 28781108 PMC5831666

[B7] Aviles-Olmos I. Dickson J. Kefalopoulou Z. Djamshidian A. Ell P. Soderlund T. et al. (2013). Exenatide and the treatment of patients with Parkinson’s disease. *J. Clin. Invest.* 123 2730–2736. 10.1172/JCI68295 23728174 PMC3668846

[B8] Aviles-Olmos I. Dickson J. Kefalopoulou Z. Djamshidian A. Kahan J. Ell P. et al. (2014). Motor and cognitive advantages persist 12 months after exenatide exposure in Parkinson’s disease. *J. Parkinsons Dis.* 4 337–344. 10.3233/JPD-140364 24662192

[B9] Baggio L. Drucker D. (2007). Biology of incretins: GLP-1 and GIP. *Gastroenterology* 132 2131–2157. 10.1053/j.gastro.2007.03.054 17498508

[B10] Barnett A. (2011). Lixisenatide: Evidence for its potential use in the treatment of type 2 diabetes. *Core Evid.* 6 67–79. 10.2147/CE.S15525 22022289 PMC3195668

[B11] Batista A. Forny-Germano L. Clarke J. Lyra E Silva N. M. Brito-Moreira J. Boehnke S. E. et al. (2018). The diabetes drug liraglutide reverses cognitive impairment in mice and attenuates insulin receptor and synaptic pathology in a non-human primate model of Alzheimer’s disease. *J. Pathol.* 245 85–100. 10.1002/path.5056 29435980 PMC5947670

[B12] Cai H. Hölscher C. Yue X. Zhang S. Wang X. Qiao F. et al. (2014). Lixisenatide rescues spatial memory and synaptic plasticity from amyloid β protein-induced impairments in rats. *Neuroscience* 277 6–13. 10.1016/j.neuroscience.2014.02.022 24583037

[B13] Cai H. Wang Z. Hölscher C. Yuan L. Zhang J. Sun P. et al. (2017). Lixisenatide attenuates the detrimental effects of amyloid β protein on spatial working memory and hippocampal neurons in rats. *Behav. Brain Res.* 318 28–35. 10.1016/j.bbr.2016.10.033 27776993

[B14] Chahine L. Lafontant D. Choi S. Iwaki H. Blauwendraat C. Singleton A. et al. (2025). LRRK2-associated parkinsonism with and without in vivo evidence of alpha-synuclein aggregates: Longitudinal clinical and biomarker characterization. *Brain Commun.* 7:fcaf103. 10.1093/braincomms/fcaf103 40114783 PMC11925012

[B15] Chavda V. Ajabiya J. Teli D. Bojarska J. Apostolopoulos V. (2022). Tirzepatide, a new era of dual-targeted treatment for diabetes and obesity: A mini-review. *Molecules* 27:4315. 10.3390/molecules27134315 35807558 PMC9268041

[B16] Christensen M. Sparre-Ulrich A. Hartmann B. Grevstad U. Rosenkilde M. Holst J. et al. (2015). Transfer of liraglutide from blood to cerebrospinal fluid is minimal in patients with type 2 diabetes. *Int. J. Obes.* 39 1651–1654. 10.1038/ijo.2015.136 26228460

[B17] Cork S. Richards J. Holt M. Gribble F. Reimann F. Trapp S. (2015). Distribution and characterisation of Glucagon-like peptide-1 receptor expressing cells in the mouse brain. *Mol. Metab.* 4 718–731. 10.1016/j.molmet.2015.07.008 26500843 PMC4588458

[B18] Cummings J. Atri A. Sano M. Zetterberg H. Scheltens P. Knop F. et al. (2026). Efficacy and safety of oral semaglutide 14 mg (flexible dose) in early-stage symptomatic Alzheimer’s disease (evoke and evoke+): Two phase 3, randomised, placebo-controlled trials. *Lancet* 407 2167–2179. 10.1016/S0140-6736(26)00459-9 41865758

[B19] de la Fuente-Fernández R. Stoessl A. J. (2002). The placebo effect in Parkinson’s disease. *Trends Neurosci.* 25 302–306. 10.1016/s0166-2236(02)02181-1 12086748

[B20] Dei Cas A. Micheli M. Aldigeri R. Gardini S. Ferrari-Pellegrini F. Perini M. et al. (2024). Long-acting exenatide does not prevent cognitive decline in mild cognitive impairment: A proof-of-concept clinical trial. *J. Endocrinol. Invest.* 47 2339–2349. 10.1007/s40618-024-02320-7 38565814 PMC11368991

[B21] Edison P. Femminella G. Ritchie C. Nowell J. Holmes C. Walker Z. et al. (2026). Liraglutide in mild to moderate Alzheimer’s disease: A phase 2b clinical trial. *Nat. Med.* 32 353–361. 10.1038/s41591-025-04106-7 41326666 PMC12823385

[B22] Eitan E. Hölscher I. L. Connell C. Thomsen M. (2026). *Evaluating KP405, a CNS -Penetrating GLP1/GIP Analogue, in a Randomized, Placebo-Controlled Phase I Study in Healthy Elderly Participants and Parkinson’s Disease Patients with Exploratory Analysis of Biomarkers in Brain-Derived Extracellular Vesicles.* Seoul: International Congress of Parkinson’s Disease and Movement Disorders.

[B23] Feng P. Zhang X. Li D. Ji C. Yuan Z. Wang R. et al. (2018). Two novel dual GLP-1/GIP receptor agonists are neuroprotective in the MPTP mouse model of Parkinson’s disease. *Neuropharmacology* 133 385–394. 10.1016/j.neuropharm.2018.02.012 29462693

[B24] Finan B. Yang B. Ottaway N. Smiley D. Ma T. Clemmensen C. et al. (2015). A rationally designed monomeric peptide triagonist corrects obesity and diabetes in rodents. *Nat. Med.* 21 27–36. 10.1038/nm.3761 25485909

[B25] Foltynie T. (2025). “Exenatide as a disease modifying treatment for parkinson’s disease- a randomised trial,” in *Proceedings of the AD/PD Conference*, (Vienna).

[B26] Forny Germano L. Koehler J. Baggio L. Cui F. Wong C. Rittig N. et al. (2024). The GLP-1 medicines semaglutide and tirzepatide do not alter disease-related pathology, behaviour or cognitive function in 5XFAD and APP/PS1 mice. *Mol. Metab.* 89:102019. 10.1016/j.molmet.2024.102019 39216535 PMC11408156

[B27] Gault V. Kerr B. Irwin N. Flatt P. R. (2008). C-terminal mini-PEGylation of glucose-dependent insulinotropic polypeptide exhibits metabolic stability and improved glucose homeostasis in dietary-induced diabetes. *Biochem. Pharmacol.* 75 2325–2333. 10.1016/j.bcp.2008.03.011 18455149

[B28] Gejl M. Gjedde A. Egefjord L. Møller A. Hansen S. Vang K. et al. (2016). In Alzheimer’s disease, 6-month treatment with GLP-1 analog prevents decline of brain glucose metabolism: Randomized, placebo-controlled, double-blind clinical trial. *Front. Aging Neurosci.* 8:108. 10.3389/fnagi.2016.00108 27252647 PMC4877513

[B29] González C. Beruto V. Keller G. Santoro S. Di Girolamo G. (2006). Investigational treatments for Type 2 diabetes mellitus: Exenatide and liraglutide. *Expert Opin. Investig. Drugs* 15 887–895. 10.1517/13543784.15.8.887 16859392

[B30] Han W. Hölscher C. Yuan L. Yang W. Wang X. Wu M. et al. (2013). Liraglutide protects against amyloid-β protein-induced impairment of spatial learning and memory in rats. *Neurobiol. Aging* 34 576–588. 10.1016/j.neurobiolaging.2012.04.009 22592020

[B31] Henry R. Rosenstock J. Logan D. Alessi T. Luskey K. Baron M. (2014). Continuous subcutaneous delivery of exenatide via ITCA 650 leads to sustained glycemic control and weight loss for 48 weeks in metformin-treated subjects with type 2 diabetes. *J. Diabetes Complications* 28 393–398. 10.1016/j.jdiacomp.2013.12.009 24631129

[B32] Hervé F. Ghinea N. Scherrmann J. M. (2008). CNS delivery via adsorptive transcytosis. *AAPS J.* 10 455–472. 10.1208/s12248-008-9055-2 18726697 PMC2761699

[B33] Hogg E. Wu T. Bresee C. Wertheimer J. Malatt C. Tan E. et al. (2023). *A Phase II, Randomized, Double-Blinded, Placebo-Controlled Trial of Liraglutide in Parkinson’s Disease.* 10.2139/ssrn.4212371

[B34] Hölscher C. (2014). Insulin, incretins and other growth factors as potential novel treatments for Alzheimer’s and Parkinson’s diseases. *Biochem. Soc. Trans.* 42 593–599. 10.1042/BST20140016 24646283

[B35] Hölscher C. (2018). Novel dual GLP-1/GIP receptor agonists show neuroprotective effects in Alzheimer’s and Parkinson’s disease models. *Neuropharmacology* 136 251–259. 10.1016/j.neuropharm.2018.01.040 29402504

[B36] Hölscher C. (2022). Glucagon-like peptide 1 and glucose-dependent insulinotropic peptide hormones and novel receptor agonists protect synapses in Alzheimer’s and Parkinson’s diseases. *Front. Synaptic Neurosci.* 14:955258. 10.3389/fnsyn.2022.955258 35965783 PMC9363704

[B37] Hölscher C. (2025). Incretin hormones GLP-1 and GIP normalize energy utilization and reduce inflammation in the brain in Alzheimer’s disease and Parkinson’s disease: From repurposed GLP-1 receptor agonists to novel dual GLP-1/GIP receptor agonists as potential disease-modifying therapies. *CNS Drugs* 39 1201–1220. 10.1007/s40263-025-01226-z 40938528 PMC12602575

[B38] Hölscher C. (2026). Semaglutide showed limited improvements in patients with Alzheimer’s disease: Revisiting the Evoke and Evoke+ clinical trials. *J. Alzheimer’s Dis.* 10.1177/13872877261458402 Online ahead of print. 42261705

[B39] Hunter K. Hölscher C. (2012). Drugs developed to treat diabetes, liraglutide and lixisenatide, cross the blood brain barrier and enhance neurogenesis. *BMC Neurosci*. 13:33. 10.1186/1471-2202-13-33 22443187 PMC3352246

[B40] Isacson R. Nielsen E. Dannaeus K. Bertilsson G. Patrone C. Zachrisson O. et al. (2011). The glucagon-like peptide 1 receptor agonist exendin-4 improves reference memory performance and decreases immobility in the forced swim test. *Eur. J. Pharmacol.* 650 249–255. 10.1016/j.ejphar.2010.10.008 20951130

[B41] Iwai T. Ito S. Tanimitsu K. Udagawa S. Oka J. (2006). Glucagon-like peptide-1 inhibits LPS-induced IL-1beta production in cultured rat astrocytes. *Neurosci. Res.* 55 352–360. 10.1016/j.neures.2006.04.008 16720054

[B42] Jerlhag E. (2018). GLP-1 signaling and alcohol-mediated behaviors; preclinical and clinical evidence. *Neuropharmacology* 136 343–349. 10.1016/j.neuropharm.2018.01.013 29337226

[B43] Johannsen P. Bentsen M. Belmont-Rausch D. Carstensen L. Eppesen R. Martin G. et al. (2025). *Semaglutide Concentration in Cerebrospinal Fluid from Patients with Early Alzheimer’s Disease After 12 Weeks of Subcutaneous Treatment.* San Diego: CTAD.

[B44] Kappe C. Tracy L. Patrone C. Iverfeldt K. Sjöholm Å (2012). GLP-1 secretion by microglial cells and decreased CNS expression in obesity. *J. Neuroinflammation* 9:276. 10.1186/1742-2094-9-276 23259618 PMC3546916

[B45] Kastin A. Akerstrom V. Pan W. (2002). Interactions of glucagon-like peptide-1 (GLP-1) with the blood-brain barrier. *J. Mol. Neurosci.* 18 7–14. 10.1385/JMN:18:1-2:07 11931352

[B46] Kastin A. Akerstrom V. (2003). Entry of exendin-4 into brain is rapid but may be limited at high doses. *Int. J. Obes. Relat. Metab. Disord.* 27 313–318. 10.1038/sj.ijo.0802206 12629557

[B47] Kim S. Moon M. Park S. (2009). Exendin-4 protects dopaminergic neurons by inhibition of microglial activation and matrix metalloproteinase-3 expression in an animal model of Parkinson’s disease. *J. Endocrinol.* 202 431–439. 10.1677/JOE-09-0132 19570816

[B48] Knudsen L. Lau J. (2019). The discovery and development of liraglutide and semaglutide. *Front. Endocrinol.* 10:155. 10.3389/fendo.2019.00155 31031702 PMC6474072

[B49] Kopp K. Glotfelty E. Li Y. Greig N. (2022). Glucagon-like peptide-1 (GLP-1) receptor agonists and neuroinflammation: Implications for neurodegenerative disease treatment. *Pharmacol. Res.* 186:106550. 10.1016/j.phrs.2022.106550 36372278 PMC9712272

[B50] Larsen P. Holst J. (2005). Glucagon-related peptide 1 (GLP-1): Hormone and neurotransmitter. *Regul. Pept.* 128 97–107. 10.1016/j.regpep.2004.08.026 15780429

[B51] Li Y. Duffy K. Ottinger M. Ray B. Bailey J. Holloway H. et al. (2010). GLP-1 receptor stimulation reduces amyloid-beta peptide accumulation and cytotoxicity in cellular and animal models of Alzheimer’s disease. *J. Alzheimers Dis.* 19 1205–1219. 10.3233/JAD-2010-1314 20308787 PMC2948479

[B52] Li Y. Perry T. Kindy M. Harvey B. Tweedie D. Holloway H. et al. (2009). GLP-1 receptor stimulation preserves primary cortical and dopaminergic neurons in cellular and rodent models of stroke and Parkinsonism. *Proc. Natl. Acad. Sci. U S A.* 106 1285–1290. 10.1073/pnas.0806720106 19164583 PMC2633544

[B53] Lingyu Z. Peng F. Wanting Z. Qianqian J. Zhang Z. Bai B. et al. (2026). The novel GLP-1/GIP dual receptor agonist DA5-CH is superior to tirzepatide and exendin-4 in the 6-OHDA Parkinson rat model. *Front. Endocrinol.* 17:1825379. 10.3389/fendo.2026.1825379 42165019 PMC13183531

[B54] Liu L. Venkatraman S. Yang Y. Guo K. Lu J. He B. et al. (2008). Polymeric micelles anchored with TAT for delivery of antibiotics across the blood-brain barrier. *Biopolymers* 90 617–623. 10.1002/bip.20998 18412128

[B55] Liu W. Jalewa J. Sharma M. Li G. Li L. Hölscher C. (2015). Neuroprotective effects of lixisenatide and liraglutide in the 1-methyl-4-phenyl-1,2,3,6-tetrahydropyridine mouse model of Parkinson’s disease. *Neuroscience* 303 42–50. 10.1016/j.neuroscience.2015.06.054 26141845

[B56] Llebaria G. Pagonabarraga J. Kulisevsky J. García-Sánchez C. Pascual-Sedano B. Gironell A. et al. (2008). Cut-off score of the Mattis dementia rating scale for screening dementia in Parkinson’s disease. *Mov. Disord.* 23 1546–1550. 10.1002/mds.22173 18546326

[B57] Long-Smith C. Manning S. McClean P. Coakley M. O’Halloran D. Holscher C. et al. (2013). The diabetes drug liraglutide ameliorates aberrant insulin receptor localisation and signalling in parallel with decreasing both amyloid-β plaque and glial pathology in a mouse model of Alzheimer’s disease. *Neuromol. Med.* 15 102–114. 10.1007/s12017-012-8199-5 23011726

[B58] Lv M. Xue G. Cheng H. Meng P. Lian X. Hölscher C. et al. (2021). The GLP-1/GIP dual-receptor agonist DA5-CH inhibits the NF-κB inflammatory pathway in the MPTP mouse model of Parkinson’s disease more effectively than the GLP-1 single-receptor agonist NLY01. *Brain Behav.* 11:e2231. 10.1002/brb3.2231 34125470 PMC8413783

[B59] Mansur R. Ahmed J. Cha D. Woldeyohannes H. Subramaniapillai M. Lovshin J. et al. (2017a). Liraglutide promotes improvements in objective measures of cognitive dysfunction in individuals with mood disorders: A pilot, open-label study. *J. Affect. Disord.* 207 114–120. 10.1016/j.jad.2016.09.056 27721184

[B60] Mansur R. Zugman A. Ahmed J. Cha D. Subramaniapillai M. Lee Y. et al. (2017b). Treatment with a GLP-1R agonist over four weeks promotes weight loss-moderated changes in frontal-striatal brain structures in individuals with mood disorders. *Eur. Neuropsychopharmacol.* 27 1153–1162. 10.1016/j.euroneuro.2017.08.433 28867303

[B61] Marquez-Meneses J. Olaya-Bonilla S. Barrera-Carreño S. Tibaduiza-Arévalo L. Forero-Cárdenas S. Carrillo-Vaca L. et al. (2025). GLP-1 analogues in the neurobiology of addiction: Translational insights and therapeutic perspectives. *Int. J. Mol. Sci.* 26:5338. 10.3390/ijms26115338 40508146 PMC12155186

[B62] Maskery M. Goulding E. Gengler S. Melchiorsen J. Rosenkilde M. Hölscher C. (2020). The dual GLP-1/GIP receptor agonist DA4-JC shows superior protective properties compared to the GLP-1 analogue liraglutide in the APP/PS1 mouse model of Alzheimer’s disease. *Am. J. Alzheimers Dis. Other Demen.* 35:1533317520953041. 10.1177/1533317520953041 32959677 PMC10623903

[B63] Matteau E. Dupré N. Langlois M. Jean L. Thivierge S. Provencher P. et al. (2011). Mattis dementia rating scale 2: Screening for MCI and dementia. *Am. J. Alzheimers Dis. Other Demen.* 26 389–398. 10.1177/1533317511412046 21697143 PMC10845364

[B64] McClean P. Hölscher C. (2014a). Liraglutide can reverse memory impairment, synaptic loss and reduce plaque load in aged APP/PS1 mice, a model of Alzheimer’s disease. *Neuropharmacology* 76 57–67. 10.1016/j.neuropharm.2013.08.005 23973293

[B65] McClean P. Hölscher C. (2014b). Lixisenatide, a drug developed to treat type 2 diabetes, shows neuroprotective effects in a mouse model of Alzheimer’s disease. *Neuropharmacology* 86 241–258. 10.1016/j.neuropharm.2014.07.015 25107586

[B66] McClean P. Parthsarathy V. Faivre E. Hölscher C. (2011). The diabetes drug liraglutide prevents degenerative processes in a mouse model of Alzheimer’s disease. *J. Neurosci.* 31 6587–6594. 10.1523/JNEUROSCI.0529-11.2011 21525299 PMC6622662

[B67] McGarry A. Rosanbalm S. Leinonen M. Olanow C. To D. Bell A. et al. (2024). Safety, tolerability, and efficacy of NLY01 in early untreated Parkinson’s disease: A randomised, double-blind, placebo-controlled trial. *Lancet Neurol.* 23 37–45. 10.1016/S1474-4422(23)00378-2 38101901

[B68] Meissner W. Remy P. Giordana C. Maltête D. Derkinderen P. Houéto J. et al. (2024). Trial of lixisenatide in early Parkinson’s disease. *N. Engl. J. Med.* 390 1176–1185. 10.1056/NEJMoa2312323 38598572

[B69] Müller T. Adriaenssens A. Ahrén B. Blüher M. Birkenfeld A. Campbell J. et al. (2025). Glucose-dependent insulinotropic polypeptide (GIP). *Mol. Metab.* 95:102118. 10.1016/j.molmet.2025.102118 40024571 PMC11931254

[B70] Müller T. Finan B. Bloom S. D’Alessio D. Drucker D. Flatt P. et al. (2019). Glucagon-like peptide 1 (GLP-1). *Mol. Metab.* 30 72–130. 10.1016/j.molmet.2019.09.010 31767182 PMC6812410

[B71] Mullins R. Mustapic M. Chia C. Carlson O. Gulyani S. Tran J. et al. (2019). A pilot study of exenatide actions in Alzheimer’s disease. *Curr. Alzheimer Res.* 16 741–752. 10.2174/1567205016666190913155950 31518224 PMC7476877

[B72] Nyberg J. Anderson M. Meister B. Alborn A. Ström A. Brederlau A. et al. (2005). Glucose-dependent insulinotropic polypeptide is expressed in adult hippocampus and induces progenitor cell proliferation. *J. Neurosci.* 25 1816–1825. 10.1523/JNEUROSCI.4920-04.2005 15716418 PMC6725940

[B73] Nyberg J. Jacobsson C. Anderson M. Eriksson P. (2007). Immunohistochemical distribution of glucose-dependent insulinotropic polypeptide in the adult rat brain. *J. Neurosci. Res.* 85 2099–2119. 10.1002/jnr.21349 17510976

[B74] Ohshima R. (2015). Age-related decrease in glucagon-like Peptide-1 in mouse prefrontal cortex but not in hippocampus despite the preservation of its receptor. *Am. J. BioSci.* 3:11. 10.11648/j.ajbio.20150301.13

[B75] Park J. Kam T. Lee S. Park H. Oh Y. Kwon S. et al. (2021). Blocking microglial activation of reactive astrocytes is neuroprotective in models of Alzheimer’s disease. *Acta Neuropathol. Commun.* 9:78. 10.1186/s40478-021-01180-z 33902708 PMC8074239

[B76] Parthsarathy V. Hölscher C. (2013). Chronic treatment with the GLP1 analogue liraglutide increases cell proliferation and differentiation into neurons in an AD mouse model. *PLoS One* 8:e58784. 10.1371/journal.pone.0058784 23536825 PMC3594148

[B77] Perry T. Greig N. (2003). The glucagon-like peptides: A double-edged therapeutic sword? *Trends Pharmacol. Sci.* 24 377–383. 10.1016/S0165-6147(03)00160-3 12871671

[B78] Reich N. Hölscher C. (2022). The neuroprotective effects of glucagon-like peptide 1 in Alzheimer’s and Parkinson’s disease: An in-depth review. *Front. Neurosci.* 16:970925. 10.3389/fnins.2022.970925 36117625 PMC9475012

[B79] Rhea E. Babin A. Thomas P. Omer M. Weaver R. Hansen K. et al. (2024). Brain uptake pharmacokinetics of albiglutide, dulaglutide, tirzepatide, and DA5-CH in the search for new treatments of Alzheimer’s and Parkinson’s diseases. *Tissue Barriers* 12:2292461. 10.1080/21688370.2023.2292461 38095516 PMC11583597

[B80] Roy A. Dawson V. Dawson T. (2025). From metabolism to mind: The expanding role of the GLP-1 receptor in neurotherapeutics. *Neurotherapeutics* 22:e00712. 10.1016/j.neurot.2025.e00712 40738791 PMC12491786

[B81] Russo S. Nestler E. (2013). The brain reward circuitry in mood disorders. *Nat. Rev. Neurosci.* 14 609–625. 10.1038/nrn3381 23942470 PMC3867253

[B82] Salameh T. Rhea E. Talbot K. Banks W. (2020). Brain uptake pharmacokinetics of incretin receptor agonists showing promise as Alzheimer’s and Parkinson’s disease therapeutics. *Biochem. Pharmacol.* 180:114187. 10.1016/j.bcp.2020.114187 32755557 PMC7606641

[B83] Trapp S. Cork S. C. (2015). PPG neurons of the lower brain stem and their role in brain GLP-1 receptor activation. *Am. J. Physiol. Regul. Integr. Comp. Physiol.* 309 R795–R804. 10.1152/ajpregu.00333.2015 26290108 PMC4666945

[B84] van Bloemendaal L. Veltman D. Ten Kulve J. Groot P. Ruhé H. Barkhof F. et al. (2015). Brain reward-system activation in response to anticipation and consumption of palatable food is altered by glucagon-like peptide-1 receptor activation in humans. *Diabetes Obes. Metab.* 17 878–886. 10.1111/dom.12506 26094857

[B85] Vijiaratnam N. Girges C. Auld G. McComish R. King A. Skene S. et al. (2025). Exenatide once a week versus placebo as a potential disease-modifying treatment for people with Parkinson’s disease in the UK: A phase 3, multicentre, double-blind, parallel-group, randomised, placebo-controlled trial. *Lancet* 405 627–636. 10.1016/S0140-6736(24)02808-3 39919773

[B86] Vrang N. Larsen P. (2010). Preproglucagon derived peptides GLP-1, GLP-2 and oxyntomodulin in the CNS: Role of peripherally secreted and centrally produced peptides. *Prog. Neurobiol.* 92 442–462. 10.1016/j.pneurobio.2010.07.003 20638440

[B87] Wang W. Davis P. Qi X. Gurney M. Perry G. Volkow N. et al. (2025). Associations of semaglutide with Alzheimer’s disease-related dementias in patients with type 2 diabetes: A real-world target trial emulation study. *J. Alzheimers Dis.* 106 1509–1522. 10.1177/13872877251351329 40552638 PMC12262134

[B88] Wang W. Wang Q. Qi X. Gurney M. Perry G. Volkow N. et al. (2024). Associations of semaglutide with first-time diagnosis of Alzheimer’s disease in patients with type 2 diabetes: Target trial emulation using nationwide real-world data in the US. *Alzheimers Dement.* 20 8661–8672. 10.1002/alz.14313 39445596 PMC11667504

[B89] Xie Y. Choi T. Al-Aly Z. (2025). Mapping the effectiveness and risks of GLP-1 receptor agonists. *Nat. Med.* 31 951–962. 10.1038/s41591-024-03412-w 39833406

[B90] Yang X. Feng P. Ji R. Ren Y. Wei W. Hölscher C. (2022). Therapeutic application of GLP-1 and GIP receptor agonists in Parkinson’s disease. *Expert Opin. Ther Targets* 26 445–460. 10.1080/14728222.2022.2079492 35584372

[B91] Yuan Z. Li D. Feng P. Xue G. Ji C. Li G. et al. (2017). A novel GLP-1/GIP dual agonist is more effective than liraglutide in reducing inflammation and enhancing GDNF release in the MPTP mouse model of Parkinson’s disease. *Eur. J. Pharmacol.* 812 82–90. 10.1016/j.ejphar.2017.06.029 28666800

[B92] Yun S. Kam T. Panicker N. Kim S. Oh Y. Park J. et al. (2018). Block of A1 astrocyte conversion by microglia is neuroprotective in models of Parkinson’s disease. *Nat. Med.* 24 931–938. 10.1038/s41591-018-0051-5 29892066 PMC6039259

[B93] Zhang L. Li C. Zhang Z. Zhang Z. Jin Q. Li L. et al. (2022). DA5-CH and semaglutide protect against neurodegeneration and reduce α-Synuclein levels in the 6-OHDA Parkinson’s disease rat model. *Parkinsons Dis.* 2022:1428817. 10.1155/2022/1428817 36419409 PMC9678466

[B94] Zhang L. Zhang L. Li Y. Li L. Melchiorsen J. Rosenkilde M. et al. (2020). The novel dual GLP-1/GIP receptor agonist DA-CH5 is superior to single GLP-1 receptor agonists in the MPTP model of Parkinson’s disease. *J. Parkinsons Dis.* 10 523–542. 10.3233/JPD-191768 31958096

[B95] Zhang Z. Hölscher C. (2020). GIP has neuroprotective effects in Alzheimer and Parkinson’s disease models. *Peptides* 125:170184. 10.1016/j.peptides.2019.170184 31705913

